# Minutes of the Michigan Dental Association

**Published:** 1883-06

**Authors:** 


### PROCEEDINGS.

### Minutes of Michigan Dental Association, Detroit, March 28, 1883.

The twenty-seventh annual meeting of the Michigan Dental Association convened in the office of Dr. G. L. Field. The meeting was called to order by the President at 8 o'clock P.M.

There being no applications for membership, the payment of dues was in order.

On motion the calling of the roll, by the Treasurer, was dispensed with until next session.

On motion of Dr. Dorrance, Detroit was decided on as the next place of meeting, to begin on the evening of the last Wednesday in March, 1884.

On motion of Dr. Field, the election of officers was postponed until to-morrow afternoon at 4 o'clock.

A letter was then read by the President from Dr. Thos. Perry, tendering his resignation as a member of the association. On motion of Dr. J. A. Robinson the letter was placed on file and his resignation accepted.

On motion of Dr. Field it was decided to employ a stenographer for the annual meeting.

On motion, adjourned.

MORNING SESSION.—THURSDAY, MARCH 29.

Meeting called to order by the President in Abstract Hall, at 10 A.M.

Dr. Land presented the name of H. A. Austin, of Detroit, for membership. Referred to the Board of Censors.

Chairman of Board of Censors announced no report to be made.

Remarks on the reception of members by Drs. Land, Field, Moore and Shattuck, all favoring a high standard.

Under report of special committee, Dr. Thomas reported a debt incurred by the banquet to the amount of $90, which was ordered paid.

Under miscellaneous business, Dr. Clawson moved a reconsideration of the vote by which it was decided to employ a stenographer. Carried.

Remarks by Drs. Clawson, Dorrance and the President.

The motion was then put as at last meeting, on employment of stenographer, which was lost.

On motion of Dr. Dorrance, Dr. French, of Pittsburgh, being present was accorded the privileges of the meeting.

The President heartily welcoming Dr. French, accorded him all privileges of the members of the society. Dr. French thanked the President and the society for their kind consideration, etc.

A letter was read by Dr. Thomas fròm Dr. Waye, of Sandusky, Ohio, in regard to the formation with Ohio of an Inter-State Society.

Motion by Dr. Thomas, that a committee of five persons be appointed by the President to attend the coming meeting of the N. O. D. A., at Sandusky, May 9 and 10, to assist in the formation of an Inter-State Society. Carried.

Remarks on the same by Drs. Taft, Thomas, Field, Harroun and others

Motion by Dr. Field that the society go into executive session. Carried.

EXECUTIVE SESSION.—THURSDAY MORNING, MARCH 29.

Meeting called to order by the President.

Remarks by Drs. Clawson, Thomas and Robinson in regard to steps to be taken to further the interest of the dental bill in the legislature.

On motion of Dr. Thomas the present business was postponed to hear Regent Duffield.

Dr. Duffield being introduced made some remarks on the advisability of extension of the course in the Dental Department of the University, speaking in a very complimentary manner on the work done by that Department, etc.

The President, in behalf of the society, extended thanks to Dr. Duffield for his suggestions, etc.

Motion by Dr. Thomas, that a fund be raised by contribution to pay the expense of a competent person to go to Lansing in the interest of the Dental Bill.

Amendment by Dr. Field, that a committee of three persons be appointed to select and recommend some person.

Amendment by Dr Lathrop, that an assessment of $1 on all members be levied to raise the required sum.

Motion and amendments put and carried.

Remarks on the same by Drs. Thomas, Clawson and Shattuck.

Motion by Dr. Thomas, that a committee of three persons be appointed by the President, in case the bill passes, to advise the Governor and assist him in the selection of a Board of Censors.

Amendment by Dr. Parker, that the Executive Committee be authorized to advise the Governor if called upon in relation to appointments.

Motion and amendment carried.

On motion of Dr. Shattuck, Regent Duffield's remarks and suggestions were referred to the same committee.

The committee appointed by the President was Drs. Thomas, Field and Lathrop.

Resolutions offered by Dr. Dorrance were referred to the Publishing Committee.

On motion, adjourned until 2 o'clock.

AFTERNOON SESSION.—MARCH 29.

Meeting was called to order by the President.

No applications for membership.

In absence of Dr. Harris, a member of the Board of Censors, the President appointed Dr. Moore to act in his place.

The President appointed the following committee to go to Sandusky: Drs. J. Lathrop, G. W. Stone, J. A. Robinson, G. L. Field, H. K. Lathrop, F. W. Clawson, G. R. Thomas, H. Cowie and W. H. Dorrance.

No report from the special committee.

Under miscellaneous business, Dr. Watling presented some water from an artesian well at Ypsilanti, recommending its virtues as an antiseptic disinfectant, etc.

The treatment of "Deciduous Teeth" passed without any paper being presented.

Dr. Robinson read a paper on "Effects and Causes," but the ideas of the author so coincided with the views of the others that it was allowed to pass without discussion.

Dr. Cowie then read a paper on the "Degeneration of the Teeth," which brought forth a very able discussion by Drs. Robinson, Parker and Land.

Dr. Thomas, as Chairman of the committee of three appointed to select some competent person to go to Lansing and work in the interest of the Dental Bill, moved that the President, Dr. A T. Metcalf, be requested to go to Lansing and act in that capacity. Carried.

The Board of Censors reported favorably upon the following names: C. B. McAuley, D.D.S., E. Saginaw, Mich.; C. B. Blackmarr, D.D.S., Jackson, Mich.; E. H. Conway, St. Clair, Mich.; D. E. Peterson, D.D.S., Waterloo, N. Y.

On motion, the rules were now suspended for the reception of dues.

The meeting was called to order by the President for the election of officers.

Drs. Clawson, Blackmarr and Miller were appointed tellers. The following were elected: Dr. W. H. Dorrance, Ann Arbor, President; Dr. F. W. Clawson, Detroit, First Vice President; Dr. U. D. Billmeyer, Ann Arbor, Second Vice President; Dr. J. B. McGregor, Port Huron, Secretary; and Dr. J. Lathrop, Detroit, Treasurer. Dr. G. R. Thomas, Detroit, was elected to fill the vacancy in the Board of Censors, Dr. Shattuck retiring.

Motion by Dr. Watling, that the incoming President appoint a University Visiting and Consulting Committee. Carried.

Chairman of the U. V. and C. Committee asked for more time in which to report, which was granted.

Very interesting remarks were now made on the method of mounting artificial crowns on natural roots by Drs. Moore, Watling, Taft, Shattuck, Harroun and Land.

Motion that further remarks be postponed until evening session. Carried.

On motion, adjourned until 7:30 P.M.

EVENING SESSION.—MARCH 29.

The meeting was called to order by the President in Abstract Parlors.

Continuation of remarks on mounting of artificial crowns upon natural roots by Drs. Taft, Shattuck and Wilson.

On suggestion of Dr. Watling, the subject of "Dental Education" was postponed until the morning session.

No papers presented on "Transplanting and Replanting Teeth."

Under the head of "Oral Surgery" some interesting cases were cited by Drs. Harroun, Shattuck and others, and the remarks and discussions on the same were intensely interesting.

Dr. Taft advised a more complete study of oral surgery by members of the profession, with the idea of making it a specialty, to whom might be referred many cases of oral surgery and treatment.

Dr. Field, Chairman of the U. V. and C. Committee, read the following report:

MR. PRESIDENT AND GENTLEMEN.—The members of the University Committee beg leave to report as follows:

On March the 15th, we visited our Dental School, at Ann Arbor, for the purpose of inspecting such of the students' work as the professors there should be able to bring to our notice, and also to make a careful inspection of the general workings of the school in its different departments as was possible in so short a time. And we are gratified to report that the Michigan Dental Association may well be pleased with its baby.

In the operative department, we saw quite a good deal of work that was not only *good* but really excellent; such work as any one might well be proud of, a result that never *could* have been attained with anything less than the closest attention to studies, and hard work, directed by earnest and competent teachers. Everything in this department seemed to move off quietly and orderly; everything seemed neat and clean; and altogether a very favorable impression was made upon the visitors.

A lecture to the graduating class by the Dean of the school, Prof. Taft, was listened to with not only pleasure, but profit, by your committee, who could not but envy these young men the privileges they enjoyed.

Descending to the laboratory, we made an inspection of the work being done there; but were not as satisfied with what we saw here, as above stairs, as there was no work *completed* for us to examine. This is the home of the *juniors*, and of course is filled with a lot of young men just passing through the first rudimental stages of dental instruction, and which is a much more difficult class to manage and teach than one that has made further advancement. We found the students here busily engaged in constructing full upper metallic plates; an outer rim being soldered to the plate, and then the teeth ground and fitted to pass up *under* this rim, then backed up and soldered. Some of these cases were very well done; that is, for young men who have had so limited a time for preparation. Other cases were poorer, but it seemed to your committee that the teaching to make such plates was not the best thing to do. It is a style of work that was done by operators thirty and forty years ago; and we think seldom done now. If it ever *is* done it never should be, for it is the filthiest plate a man ever wore, and is not to be compared to a rubber plate, if properly made; and your committee report that, in their judgment, the time thus expended would be better employed in teaching students to do the work that they will be called upon to do when they shall have completed their studies. It is claimed, we know, that this is taught them to initiate them into the mysteries of the blow pipe, the moulding box and die-making. *This*, of course, is very essential; but at the same time we believe that this can all be done by teaching the making of such plates as will be made by them when they are cast upon their own resources.

Say what we may, rubber work must and *will* be done until some substitute is invented that will be equally cheap and fill the bill as well or better. Then let it be taught, and then as these young men can, let them discard it from their practice, and replace it with gold, platinum, etc.

It is much to be regretted that there cannot be a larger number of teachers in the laboratory, as the two that have the whole charge of this department have more to do than should be required of them.

On motion the report was accepted.

Remarks on "Prosthetic Dentistry," by Drs. Dorrance, Field, Land, McGregor and Clawson.

Motion to adjourn until to-morrow morning at 9 o'clock, sharp. Carried.

MORNING SESSION.—MARCH 30.

The meeting was called to order by the President at 9:30 A. M.

The minutes were read.

On motion, the report was accepted.

Dr. Clawson, Chairman of the Publishing Committee, reported recommending that Dr. Dorrance's resolution lay over another year.

Dr. Taft called for the reading of the resolution.

Remarks by Drs. Dorrance, Clawson and the President.

On motion of Dr. Taft the resolutions were recommitted to committee.

Remarks by Drs. Watling and Clawson.

Regular order of business resumed, Dr. Clawson, in the chair.

On motion of Dr. Field, a committee was appointed to draft resolutions upon the death of Dr. Goddard, of Louisville. Committee—Drs. Taft, Field and Harroun.

Bills presented, audited, and paid.

Dr. Taft offered to print abstracts of the proceedings of the meeting.

Dr. Thomas called the attention of the society to the duties of the Secretary, and that he be instructed to notify persons appointed upon committees directly after the close of the meeting.

A letter was read from Mrs. Hauxhurst asking that a copy of the resolutions passed by the society upon the death of her husband be sent her.

The Secretary was so instructed.

Dr. Thomas asked that the society express their opinion upon the feasibility of forming an inter-state society.

Remarks by Drs. Taft, Field, and others.

The general opinion expressed was that it would tend to lessen the interest in the Michigan Dental Association, and if such was to be the result, it had better not be adopted.

Dr. Dorrance called attention to article 2 of the By-Laws.

On motion of Dr. Thomas, the Secretary was instructed to forward a copy of the resolutions to Mrs. Hauxhurst.

Motion of Dr. Watling, that the installation of officers take place at 4 o'clock this afternoon; was carried.

Dr. Shattuck gave notice that he would move for a change in the constitution so as to create a junior membership in the Michigan Dental Association.

Bills audited and ordered paid for postage, etc.

The President appointed the following committees:

To fill vacancies in the U. V. and C. Committee, Drs. A. T. Metcalf and J. C. Parker, also H. K. Lathrop in place of Dr. B. Bannister.

Visiting and Consulting Committee to University—Drs. G. L. Field, E. C. Moore, A. T. Metcalf, J. C. Parker and H. K. Lathrop.

Executive Committee—Drs. G. R. Thomas, E. C. Moore, H. Cowie.

Publishing Committee—Drs. G. L. Field, J. Taft, J. B. McGregor.

Dr. Field wished the society to explain the duties of the U. V. and C. Committee. Drs. Watling and Taft expressed a pleasure to meet the committee at the University at any time during the course and hoped they would make free to make any suggestions that might occur to them.

On motion, the report of the U. V. and C. Committee was accepted and placed on file.

Dr. Watling's paper on "Dental Education" was then read, and discussed by Drs. Harroun, Land, Clawson and Taft. All seemed to be of the opinion that the training in operation and manipulation should begin as soon after the literary education as possible; also of the advantage and need of increase of time with the gradual increase of curriculum, etc.

Dr. Taft moved that the final adjournment take place at 6 o'clock this evening. Carried.

Motion to adjourn till 2 o'clock. Carried.

The meeting was called to order by the President at 2:30 P.M.

The Board of Censors reported favorably on J. W. Essig for membership, who was unanimously elected.

Dr. Rowley read a paper on "Exposed Pulps and Treatment."

On motion by Dr. Taft twenty minutes were devoted to the discussion of the paper.

The paper was then discussed by Drs. French, Land, Shattuck, Taft, Rowley and Long.

Report of resolutions from committee upon Dr. Goddard's death.

On motion, the resolutions were adopted.

A copy of resolutions ordered sent by Secretary to the family of Dr. Goddard.

A paper was then read by Dr. Porter.

Paper read by Dr. Dorrance.

Bills audited and ordered paid.

TREASURER'S REPORT.

Mr. President, and Gentlemen of the Michigan State Dental Association, I herewith submit to you my report of moneys received and disbursed since my last report:

RECEIPTS.

| | |
|---|---|
| To money on hand from last year | $ 7 98 |
| " cash from new members | 10 00 |
| " " " annual dues | 118 00 |
| " " " special assessment | 38 00 |
| Total | $173 98 |

DISBURSEMENTS.

| | |
|---|---|
| By paid note held by Dr. Cowie | $74 00 |
| " " F. S. Ackerman bill | 15 00 |
| " " E. C. Moore's bill | 5 58 |
| " " I. F. Eby | 5 00 |
| | $99 58 |

| | |
|---|---|
| Receipts | $173 98 |
| Disbursements | 99 58 |
| Balance | $ 74 40 |

All of which is respectfully submitted.

J. LATHROP, Treasurer.

On motion, the Treasurer's Report was accepted.

Report from Dr. Thomas on the papers to be presented at the next meeting.

The President, Dr. Dorrance, was then conducted to the chair, with a few remarks from the retiring President, Dr. A. T. Metcalf.

On motion of Dr. Thomas, a vote of thanks was extended F. S. Ackerman & Co. Also, Mr. Caulkins, for tendering the use of the rooms to the Association.

On motion, by Dr. Clawson, a vote of thanks was given to F. S. Ackerman & Co., for publishing the transactions of last annual meeting.

Dr. Perry and Dr. French to be made honorary members at the next annual meeting.

On motion, adjourned.

Papers to be presented at next meeting, are as follows:

Prosthetic Dentistry, Dr. Dorrance; Atmospheric Dentures, Dr. Land; Diseases of Gums, Causes, and Treatment, Dr. Cleland; Oral Surgery, Dr. Case; Histology, Dr. Britton; Methods and Materials for Filling Teeth, Dr. Moore; Treatment of Deciduous Teeth, Drs. Cowie and Taft; Pulpless Teeth, Dr. Douglass; Exposed Pulps, Dr. Rowley; Dental Caries, Dr. Shattuck.

